# Progression of Coexistent Aortic Valve Disease and Its Clinical Outcome in Patients Who Underwent Mitral Valve Surgery: A Single-Centre Observational Cohort Study

**DOI:** 10.7759/cureus.110280

**Published:** 2026-06-05

**Authors:** Prabhas T Durbesula, Gautham A, Ramsankar P, Sai Chandran BV

**Affiliations:** 1 Department of Cardiothoracic and Vascular Surgery, Jawaharlal Institute of Postgraduate Medical Education and Research, Puducherry, IND

**Keywords:** aortic regurgitation, aortic stenosis, aortic valve disease, echocardiography, mitral valve surgery

## Abstract

Background: Coexistent mild aortic valve disease is frequently observed in patients undergoing mitral valve surgery, yet the clinical significance and progression of these lesions remain uncertain. This study aimed to evaluate the progression and clinical outcomes of mild aortic valve disease following mitral valve surgery.

Methods: This single-centre observational study included 80 patients who underwent primary mitral valve surgery with documented mild aortic valve disease. Patients were evaluated through a hybrid design, consisting of a retrospective cohort (n=43) from 2010 to 2017 and a prospective cohort (n=37) from 2016 to 2019, using echocardiographic and clinical assessments.

Results: At baseline, out of the 80 patients, 82.5% (n=66) presented with aortic regurgitation (AR) lesions, which comprised isolated mild AR (n=53; 66.2%), trivial AR (n=4; 5%), moderate AR (n=4; 5%), and mixed mild AR with mild aortic stenosis (AS) (n=5; 6.2%). The remaining 17.5% (n=14) presented with isolated mild AS. Follow-up clinical and echocardiographic assessment was completed in 74 patients, while six patients were lost to follow-up. During follow-up (mean duration: 3.54±1.48 years), most assessed patients remained clinically and echocardiographically stable. Among patients with isolated mild AR at baseline, eight patients progressed to moderate AR, while the remaining patients did not demonstrate clinically meaningful progression. Pressure half-time decreased from 643.53±103.90 ms preoperatively to 592.30±120.55 ms postoperatively (t=5.122; p<0.001), and vena contracta increased from 0.26±0.05 cm to 0.31±0.10 cm (t=4.472; p<0.001), without clinically significant deterioration. In patients with aortic stenosis, the aortic valve area decreased from 1.66±0.04 cm² to 1.59±0.02 cm² (t=2.047; p=0.044), and the mean gradient increased from 16±1.73 mmHg to 19±1.00 mmHg (t=2.145; p=0.035). Kaplan-Meier analysis demonstrated an event-free survival rate of 94.6% at the final follow-up. Multivariate analysis identified preoperative aortic valve lesion as the only significant predictor of disease progression (OR: 0.038; 95% CI: 0.002-0.948; p=0.046).

Conclusions: Mild aortic valve disease in patients undergoing mitral valve surgery generally remains stable and does not demonstrate significant clinical progression. Routine echocardiographic surveillance remains important to identify the minority of patients who may develop progressive valve dysfunction.

## Introduction

Aortic valve disease, including aortic stenosis (AS) and aortic regurgitation (AR), represents an important cardiovascular condition associated with substantial morbidity and mortality when clinically significant disease remains untreated [1]. Abnormalities in aortic valve structure can disrupt left ventricular outflow and systemic circulation, contributing to progressive ventricular dysfunction, heart failure, and adverse cardiovascular events [2,3]. In rheumatic valvular disease, surgical repair or replacement is commonly required when valve dysfunction becomes clinically significant, particularly in patients with advanced mitral valve pathology [4]. In patients undergoing rheumatic mitral valve replacement, coexistent mild aortic valve disease may be left untreated at the index operation, but its subsequent natural history requires postoperative surveillance [5].

Another significant cause of cardiac dysfunction and heart failure in most countries is mitral valve disease, such as mitral stenosis and mitral regurgitation [6]. Surgical repair, replacement, or valvotomy of the mitral valve is commonly carried out in severely diseased patients to reduce symptoms and improve long-term clinical outcomes [7]. Previous studies specifically evaluating mild aortic valve disease after mitral valve intervention or rheumatic mitral valve surgery have shown that these lesions may remain stable in many patients but can progress over longer follow-up, particularly when baseline AS is present [8,9].

This uncertainty creates a clinically relevant surgical question: whether mild aortic valve pathology identified at the time of mitral valve surgery should be treated concomitantly or managed conservatively with structured postoperative monitoring [5,8,9]. Although concomitant valve intervention may increase procedural complexity, available evidence suggests that selected mild aortic valve lesions can be observed safely, provided that patients undergo regular clinical and echocardiographic follow-up [5,8,9]. Thus, knowledge of mild aortic valve disease behaviour after mitral valve surgery is crucial for surgical decision-making and postoperative risk stratification.

In mitral regurgitation, altered left ventricular volume loading and abnormal transmitral flow dynamics may affect global ventricular hemodynamics after surgical correction [10,11]. After the surgical correction of mitral regurgitation, changes in left ventricular loading conditions and forward flow may influence postoperative clinical and echocardiographic assessment, although the specific effect on coexisting mild aortic valve lesions remains insufficiently defined [12]. In patients with multiple left-sided valve lesions, hemodynamic interaction between AS and concomitant mitral regurgitation may influence clinical interpretation and surgical decision-making, supporting the need for careful echocardiographic assessment and follow-up planning [10,13].

Despite such physiological considerations, the progression of mild aortic valve disease in patients who have undergone mitral valve surgery over a long period of time is not well documented. Postoperative clinical outcomes, including functional status, symptom improvement, and major adverse cardiovascular events (MACEs), also require further evaluation in this patient group. Clarifying these outcomes may help determine whether conservative management of mild aortic valve disease at the time of mitral valve surgery is appropriate or whether more aggressive concomitant intervention should be considered in selected patients.

Objectives of the study

The objective of this single-centre observational study was to evaluate the progression of coexistent mild aortic valve disease in patients who underwent primary mitral valve surgery. The primary objective was to assess whether pre-existing mild AR, AS, or mixed aortic valve disease progressed during postoperative follow-up using serial clinical and transthoracic echocardiographic assessments. Secondary objectives were to evaluate postoperative clinical outcomes, including functional status and MACEs, and to identify potential predictors of aortic valve disease progression after mitral valve surgery.

## Materials and methods

Study design and ethical approval

The Department of Cardiothoracic and Vascular Surgery at Jawaharlal Institute of Postgraduate Medical Education and Research in Puducherry, India, conducted this observational study between September 2016 and June 2019. The purpose of this study was to investigate the development of coexistent aortic valve disease and the clinical outcomes of patients who had undergone mitral valve surgery. Ethical approval was obtained from the institute's Institutional Ethics Committee (Human Studies) (approval number: JIP/IEC/2018/317). Written informed consent was obtained from all participants before their inclusion in the study.

Study population

A total of 80 patients were included in the study. The retrospective cohort consisted of 43 patients who underwent surgery between 2010 and 2017 and were followed up through clinical records and contemporary reassessments. The prospective cohort consisted of 37 patients enrolled and monitored between 2016 and 2019. All patients in both cohorts met the inclusion criteria of documented coexistent aortic valve disease at the time of their index mitral valve surgery.

The study included patients who underwent primary mitral valve surgery and had documented coexistent aortic valve disease at the time of surgery. Eligible patients had evidence of AS, AR, or mixed aortic valve disease on preoperative transthoracic echocardiography (TTE). The baseline cohort included isolated mild AR in 53 patients, trivial AR in four patients, borderline/moderate asymptomatic AR in four patients, mixed mild AR with mild AS in five patients, and isolated mild AS in 14 patients, giving a total baseline sample of 80 patients. Inclusion required complete baseline clinical and echocardiographic data and availability for attempted postoperative clinical and echocardiographic follow-up. While the study primarily focused on coexistent mild aortic valve disease, four patients (5%) with borderline/moderate asymptomatic AR at baseline were included to serve as a comparative cohort to evaluate whether baseline lesion severity accelerated postoperative progression.

The baseline evaluation for all 80 patients consisted of a comprehensive clinical assessment and standardised TTE performed within 30 days before the index mitral valve surgery. Clinical assessment included medical history, New York Heart Association (NYHA) functional class, comorbid conditions such as hypertension and diabetes mellitus, and physical examination for signs of heart failure. Echocardiographic assessment included aortic valve morphology, peak and mean transaortic gradients, aortic valve area (AVA) calculated using the continuity equation, and AR severity assessed using jet width and vena contracta. These variables served as reference values for subsequent longitudinal comparisons.

By "availability of the patient", the authors refer to successful recruitment and retention for follow-up assessment. For the retrospective cohort (2010-2017), this meant that patients could be contacted through telephonic records and were able to visit the centre for clinical and echocardiographic evaluation. For the prospective cohort, it referred to compliance with scheduled one-year and two-year follow-up protocols. Of the 80 patients included at baseline, 74 completed postoperative clinical and echocardiographic follow-up, while six patients were lost to follow-up. Therefore, baseline demographic and lesion characteristics are reported using 80 as the denominator, whereas postoperative progression and follow-up outcome analyses are reported using the assessed follow-up cohort unless otherwise specified.

Patients were excluded if they had undergone previous mitral valve surgery or reoperation, required aortic valve surgery during the index mitral valve procedure, were readmitted with prosthetic valve thrombosis, had connective tissue disorders such as Marfan syndrome, or lacked objective documentation of aortic valve disease at the time of initial mitral valve surgery.

Sample size determination

The Cochran formula was used in determining the required sample size for the estimation of proportions. By assuming an estimated progression rate of aortic valve disease based on previous studies evaluating untreated mild aortic valve disease after mitral valve intervention and rheumatic mitral valve surgery [8,9], the determined sample size was 80 patients. To compensate for potential losses in the follow-up, the recruitment stage involved a larger number of subjects. The formula used for the sample size calculation was $n=\frac{z^2 \times p(1-p)}{d^2}$ where $n$ represents the required sample size, $Z$ represents the standard normal deviate corresponding to the desired confidence level, $p$ represents the estimated proportion of the population with disease progression, and $d$ represents the absolute precision.

Data collection and follow-up

This study utilised a hybrid observational design comprising both retrospective and prospective patient cohorts. The retrospective cohort included patients who underwent mitral valve surgery between 2010 and 2017 and were identified through hospital surgical records. According to the year-wise surgical distribution, 41 patients underwent surgery between 2010 and 2015, while 39 patients underwent surgery during 2016-2017. The year-wise surgical distribution refers to the date of index mitral valve surgery, whereas retrospective or prospective classification was based on the method by which follow-up data were obtained. Patients from the overlapping transition period were classified according to whether their follow-up data were obtained retrospectively from records or prospectively through scheduled follow-up.

For patients in the retrospective cohort, follow-up data were obtained by reviewing postoperative hospital records. These patients were also invited for contemporary outpatient clinical and echocardiographic reassessment. Of the total study population, 43 patients belonged to the retrospective cohort covering the period 2010-2017, while 37 patients were enrolled in the prospective follow-up cohort during 2016-2019. This distinction explains the overlap between the surgical years and enrolment/follow-up periods.

The prospective cohort consisted of patients registered during the study period from 2016 to 2019. These patients were monitored through a pre-established follow-up programme and underwent scheduled clinical and echocardiographic assessments at one and two years after surgery. These assessments were performed to evaluate the progression of aortic valve disease and postoperative clinical outcomes.

For each patient, follow-up duration was calculated as the interval between the date of index mitral valve surgery and the date of the most recent documented clinical and echocardiographic follow-up assessment. The mean duration from surgery to follow-up evaluation was 3.54±1.48 years. Of the 80 patients initially included in the study, 74 completed follow-up assessments, while six patients were lost to follow-up. Accordingly, baseline characteristics were analysed using the full cohort of 80 patients, whereas follow-up progression and postoperative outcome analyses were based on the 74 patients with completed follow-up assessments unless explicitly stated otherwise.

Clinical and echocardiographic assessment

Clinical examination and TTE were performed in all patients to assess the severity and progression of aortic valve disease. Echocardiographic parameters were used to determine the severity of AR and AS according to current valvular heart disease guideline recommendations. Clinical assessment included patient symptoms, functional capacity, NYHA classification [14], and history of hospitalisation for MACEs. Patients in the prospective cohort underwent repeat clinical and echocardiographic evaluation during scheduled follow-up visits.

Statistical analysis

All collected data were entered into Microsoft Excel (Microsoft Corporation, Redmond, Washington, United States) and subsequently analysed using IBM SPSS Statistics for Windows, Version 28.0 (IBM Corp., Armonk, New York, United States). Continuous variables were tabulated in mean, standard deviation, and frequencies, and percentages were used to tabulate categorical variables. The paired t-test was used as the test of comparing the preoperative and postoperative echocardiographic parameters, and the chi-squared test was used to test categorical variables. The estimation of the duration of survival without event during the follow-up was done using Kaplan-Meier survival analysis. Multivariate and logistic regression analysis was carried out to determine independent predictors of the progression of aortic valve disease, and the outcomes were presented as odds ratios (OR) with respective 95% confidence intervals (CI). A p-value below 0.05 was taken to be statistically significant.

## Results

Baseline demographic characteristics

The study involved 80 patients who had coexistent aortic valve disease and were undergoing mitral valve surgery. The clinical profile and epidemiologic distribution of patients with combined valvular pathology require demographic assessment to understand them. The age and sex structure serve to gain a better understanding of the disease patterns, the factors that may be the aetiology, and the population that is most commonly affected by valvular heart disorders. Valvular diseases, especially of rheumatic pathology, in most areas were more apt to occur in rather young persons, and their occurrence was frequently different in males and in females. The assessment of these baseline characteristics makes it easier to interpret clinical outcomes in a better way and compare their findings with those that have been reported in other clinical cohorts. Detailed demographic characteristics are summarised in Table 1.

**Table 1:** Baseline demographic characteristics of the study population (n=80)

Variable	Category	Frequency (n)	Percentage (%)
Gender	Male	23	28.8
	Female	57	71.2
Age (years)	≤20	11	13.8
	21-30	9	11.2
	31-40	26	32.5
	41-50	22	27.5
	≥51	12	15.0
Mean age±SD			38.04±12.69

Distribution of mitral valve surgeries by year

**Figure 1:**
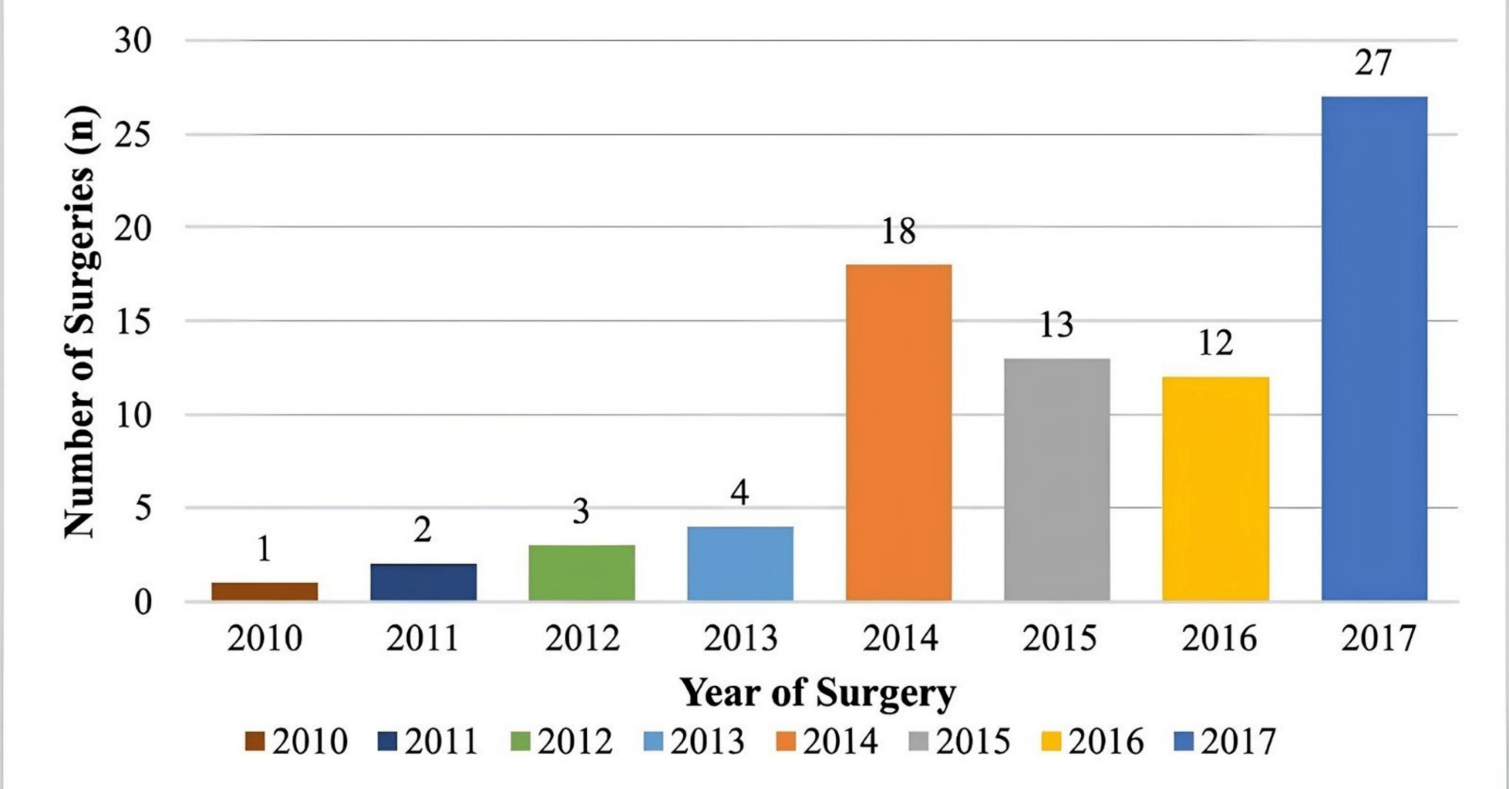
Annual distribution of mitral valve surgeries during the study period

The time-based distribution of the number of mitral valve surgeries proved that the number of procedures progressively increased in the later years of the timeframe of the study, which shows that the workload of the surgery was growing with time. The previous years brought in fewer cases as compared to the earlier years, but most of the surgeries were done during the later years of the observation period. This trend can be an indication of better referral trends, higher rates of diagnostic detection, or a higher surgical capacity over the period of the study. The average time taken between the operation and follow-up examination was 3.54 years and 1.48 years, respectively. The annual distribution of surgeries is illustrated in Figure 1.

Baseline and follow-up aortic valve lesions

The evaluation of aortic valve condition at the time of mitral valve surgery showed that AR was the most common associated lesion in the study group. At baseline, 66 of 80 patients (82.5%) had AR-related lesions. These included isolated mild AR in 53 patients (66.2%), trivial AR in four patients (5%), borderline/moderate asymptomatic AR in four patients (5%), and mixed mild AR with mild AS in five patients (6.2%). The remaining 14 patients (17.5%) had isolated mild AS. These categories account for the full baseline cohort of 80 patients.

Follow-up clinical and echocardiographic assessment was completed in 74 patients, while six patients were lost to follow-up. Therefore, postoperative lesion distribution and progression were assessed using the 74 patients with available follow-up data. In the postoperative follow-up, the majority of assessed patients remained stable, while a minority showed an increase in AR severity. Among assessed patients with isolated mild AR at baseline, eight progressed to moderate AR during follow-up, while the remaining assessed patients remained stable. Patients with trivial AR, borderline/moderate asymptomatic AR, isolated mild AS, and mixed mild AR with mild AS did not show clinically meaningful progression during the follow-up period.

Clinical outcomes and comorbidities

Postoperative assessment showed that most patients maintained good functional status following mitral valve surgery. Clinical outcome assessment was based on the 74 patients who completed postoperative follow-up, while six patients were lost to follow-up. Most assessed patients were classified as NYHA functional class I, and only a small proportion had mild functional limitation at follow-up, indicating generally preserved postoperative functional capacity.

Evaluation of comorbidities showed that most patients did not have major documented comorbid conditions, while a small minority had metabolic or cardiovascular risk factors, including diabetes mellitus or hypertension. Cardiac readmission during follow-up was uncommon and occurred mainly among patients with aortic valve disease progression or associated comorbid conditions. Overall, postoperative clinical outcomes were favourable in the assessed follow-up cohort.

Echocardiographic changes following surgery

Comparison of preoperative and postoperative echocardiographic parameters showed statistically significant shifts in the selected parameters used to assess aortic valve function. Paired sample t-test analysis demonstrated significant differences in pressure half-time, vena contracta, aortic valve area, and mean gradient, with corresponding t-values and p-values reported. AR and AS parameters exhibited measurable postoperative variation; however, the magnitude of these changes did not indicate clinically meaningful deterioration in valve function. The echocardiographic findings suggest that, despite minor hemodynamic alterations following mitral valve surgery, there was no evidence of progression to severe aortic valve disease during the follow-up period. Table 2 presents a comparison of echocardiographic measurements before and after mitral valve surgery to evaluate changes in aortic valve function.

**Table 2:** Pre- and postoperative aortic valve parameters The statistical test used was a paired-sample t-test. Exact t-statistic values are reported for all parameters. Although statistically significant postoperative changes were observed in pressure half-time, vena contracta, aortic valve area, and mean gradient, these changes did not correspond to clinically meaningful progression of aortic valve disease.

Parameter	Preoperative mean±SD	Postoperative mean±SD	Test statistic (t)	P-value	Clinical interpretation
Pressure half-time (ms)	643.53±103.90	592.30±120.55	5.122	<0.0001	No progression
Vena contracta (cm)	0.26±0.05	0.31±0.10	4.472	<0.001	No progression
Aortic valve area (cm²)	1.66±0.04	1.59±0.02	2.047	0.044	No progression
Mean gradient (mmHg)	16±1.73	19±1.00	2.145	0.035	No progression

Progression of aortic valve disease

Postoperative clinical and echocardiographic follow-up was completed in 74 of the 80 baseline patients, while six patients were lost to follow-up and excluded from progression analysis. Therefore, progression analysis was based on patients with available follow-up data. Among assessed patients with isolated mild AR at baseline, eight progressed to moderate AR, while the remaining assessed patients remained stable. Patients with baseline trivial AR, borderline/moderate asymptomatic AR, isolated mild AS, and mixed mild AR with mild AS did not show clinically meaningful progression during follow-up. No patient progressed to severe AR or severe AS during the follow-up period.

Predictors of disease progression

Multivariate analysis on logistic regression was performed to assess possible independent predictors that would help to assess the progression of aortic valve disease after mitral valve surgery. The results of the regression model indicated the overall statistical significance and acceptable predictability in identifying the risks of progression of patients. Out of the variables that were incorporated in the model, preoperative aortic valve lesion was the only variable that had a significant level of relation with the development of the disease in the future. Additional clinical and demographic conditions such as age, sex, hypertension, functional status, and MACEs were not significantly associated with progression in this cohort at an independent level. Table 3 shows the association between clinical variables and the likelihood of progression of aortic valve disease.

**Table 3:** Logistic regression analysis of the predictors of disease progression The statistical test used is a binary logistic regression. AV: aortic valve; NYHA: New York Heart Association; MACE: major adverse cardiovascular event; HTN: hypertension; DM: diabetes mellitus; OR: odds ratio; CI: confidence interval; S.E.: standard error

Variable	B	S.E.	Wald	P-value	OR	95% CI for OR
Sex	0.482	0.888	0.294	0.587	1.619	0.284-9.219
Age	-0.020	0.032	0.369	0.544	0.981	0.921-1.044
Preop AV lesion	-3.266	1.639	3.971	0.046	0.038	0.002-0.948
Preop HTN	-0.005	0.005	1.072	0.301	0.995	0.985-1.005
NYHA class MACE	-1.259	1.651	0.581	0.446	0.284	0.011-7.221
HTN_DM	0.719	1.480	0.236	0.627	2.052	0.113-37.355
MACE	-1.856	1.556	1.423	0.233	0.156	0.007-3.301

Event-free survival analysis

Kaplan-Meier survival analysis was conducted to determine event-free survival regarding MACEs. The number of events experienced within the study period was only two, making the cumulative survival probability high. The initial event took place in 2012, and the survival probability was 98.6%, while the second event took place in 2017 and had a survival probability of 94.6%. A majority of participants were censored by the conclusion of the observation period (97.2%), and this means that they were either event-free or lost to follow-up. The median survival time was not calculable as more than half of the participants were free of an event. Subgroup analysis revealed that male patients had 100% survival in terms of MACE and there were two incidences in the female patients, leading to a survival rate of 96.5%. Nevertheless, the log-rank test did not find a statistically significant difference between sexes (p=0.13). On the same note, there were no significant differences in survival rates among various age groups (log-rank test p=0.18).

## Discussion

The results show that female patients outnumbered male patients, with women forming 71.2% of the study population. The NYHA functional classification remains clinically relevant for describing symptom burden and functional limitation in patients with cardiovascular disease [14]. The average age of the participants was relatively young (38.04 years), suggesting that the valve pathology in this cohort was unlikely to be explained solely by degenerative disease, which is more common in older populations. In regions where rheumatic heart disease remains prevalent, early-onset valvular dysfunction is frequently linked to rheumatic valve pathology, while congenital and developmental mechanisms may also contribute to selected forms of aortic valve disease [15-17]. The concentration of cases in the 31-50-year age groups is therefore compatible with a non-degenerative pattern of valvular disease, particularly in populations where rheumatic valve disease remains clinically important [15,16]. Conversely, degenerative valve disease in older populations more commonly presents as calcific AS or degenerative mitral valve disease, although mixed and combined valve disease may also occur and requires careful assessment [18].

The problem of AR remains clinically relevant when patients undergo mitral valve surgery because underlying aortic valve pathology may influence long-term hemodynamics and postoperative outcomes. Previous studies have shown that chronic AR may influence clinical outcomes in patients with native aortic valve disease, while moderate AR identified at the time of surgery remains a debated issue in surgical decision-making [19,20]. Mixed and multiple valvular lesions require integrated assessment because treatment decisions depend on lesion severity, ventricular consequences, symptom status, and procedural risk [21]. The coexistence of mitral and aortic valve lesions underscores the importance of detailed echocardiographic evaluation before and after mitral valve surgery to ensure accurate diagnosis, appropriate surveillance, and timely identification of clinically relevant progression [6,21].

Improvement in functional capacity after mitral valve intervention has been reported in patients with clinically significant mitral regurgitation, supporting the functional benefit of correcting mitral valve pathology in appropriately selected patients [22]. The positive hemodynamic effect of mitral valve correction is also reflected by improvement in functional class in many postoperative cohorts. In patients with multiple valve disease, interaction between valve lesions may alter apparent severity and influence management strategy, supporting the need for longitudinal clinical and echocardiographic follow-up [23]. These observations underline the need for follow-up clinical and echocardiographic assessment in patients with concurrent valve disease after surgery.

Comorbid conditions are significant determinants of postoperative outcomes in surgical and cardiovascular populations. Postoperative complications after cardiac surgery have been associated with worse long-term survival, supporting the importance of perioperative risk assessment and postoperative surveillance in patients undergoing valve surgery [24]. In patients undergoing aortic valve interventions, metabolic dysfunction and multimorbidity have been associated with adverse cardiovascular outcomes, reinforcing the importance of comorbidity assessment in valvular heart disease populations [25]. Cardiometabolic comorbidities such as diabetes mellitus and hypertension may impair myocardial function and cardiac remodelling through adverse metabolic, inflammatory, and redox-related mechanisms [26]. Postoperative atrial fibrillation is also clinically relevant after surgical valve intervention and has been associated with adverse long-term outcomes, including mortality, stroke, thromboembolic events, heart failure hospitalisation, recurrent atrial fibrillation, and bleeding [27].

The postoperative changes in echocardiographic parameters observed in this study may reflect altered loading conditions or cardiac physiology rather than true structural progression of aortic valve disease. Effective hemodynamic assessment requires interpretation of measured parameters within the broader clinical and physiologic context rather than reliance on isolated numerical changes [28]. Advanced echocardiographic assessment can provide additional information on myocardial function, but imaging findings should still be interpreted alongside symptoms, functional status, and serial follow-up data [29]. Likewise, small changes in aortic valve area or transvalvular gradients may represent physiologic hemodynamic adaptation rather than structural deterioration of the aortic valve, and guideline-based valvular assessment requires integration of clinical findings, Doppler measurements, valve morphology, ventricular response, and longitudinal follow-up [30]. These findings support interpreting echocardiographic values in the larger clinical context, rather than acting on isolated numerical variation. Given the observational design and mid-term follow-up duration, these findings should be interpreted as evidence of short- to mid-term stability of mild coexistent aortic valve disease after mitral valve surgery, rather than definitive evidence of long-term disease behaviour.

Predictors of progression

Our multivariate logistic regression analysis demonstrated that the preoperative aortic valve lesion was the only statistically significant predictor of future disease progression (OR: 0.038; 95% CI: 0.002-0.948; p=0.046). Other factors, including age, hypertension, and sex, did not show a significant correlation with progression in this cohort.

Strengths, limitations, and future recommendations

The primary strength of this study is the long-term follow-up (mean: 3.54±1.48 years) of a specific patient group that is often overlooked in surgical planning. However, the study has several limitations. The single-centre design may limit the generalisability of the findings to larger and more diverse populations. The relatively small sample size of 80 patients may restrict the ability to identify less common predictors of progression or subtle postoperative changes. Additionally, the hybrid retrospective-prospective design may introduce inherent selection bias.

The predominance of rheumatic heart disease in the study population may also have influenced the observed patterns of valve pathology and may not fully reflect populations with a higher prevalence of degenerative valvular disease. The follow-up duration, although useful for mid-term assessment, remains limited for evaluating the long-term progression of aortic valve disease after mitral valve surgery. Interobserver variability in echocardiographic assessment is another potential limitation.

Future investigations should include larger, multicentre cohorts to improve generalisability and represent different etiologies of valvular disease. A longer follow-up is required to determine whether mild pre-existing aortic valve lesions remain stable or progress to clinically significant disease after mitral valve surgery. Separate analysis of rheumatic and non-rheumatic subgroups may help clarify whether disease behaviour differs according to underlying pathology. Postoperative assessment could be strengthened through standardised serial echocardiographic protocols, advanced imaging where appropriate, and structured clinical outcome measures. Further evaluation of predictors of progression, especially preoperative aortic valve lesions, may support risk stratification, surveillance planning, and individualised management.

## Conclusions

This study demonstrates that mitral valve surgery has minimal influence on the progression of pre-existing mild aortic valve disease in most patients. The echocardiographic analysis presented at the postoperative stage demonstrated relatively constant parameters and no significant changes in the severity of AR or AS in most of the cases. These observations indicate that minor abnormalities in aortic valves may frequently be left and not corrected during the surgery for the mitral valve. Moreover, there was a significant improvement in the symptoms in the majority of patients after an operation, which is a clear functional advantage of the mitral valve pathology repair. However, the percentage of patients who deteriorated functionally in the follow-up was low, and most of them were due to the development of AR, which demonstrates the necessity of a regular postoperative clinical and echocardiographic follow-up. Premarked abnormalities of the aortic valves in preoperative patients proved to be a significant indicator of subsequent disease development. Comprehensively, the favourable outcome of event-free survival of this cohort contributes to the safety, durability, and clinical efficacy of mitral valve repair among the right patients.
